# High-throughput marker discovery in melon using a self-designed oligo microarray

**DOI:** 10.1186/1471-2164-11-269

**Published:** 2010-04-28

**Authors:** Ron Ophir, Ravit Eshed, Rotem Harel-Beja, Galil Tzuri, Vitaly Portnoy, Yoseph Burger, Shai Uliel, Nurit Katzir, Amir Sherman

**Affiliations:** 1 Genomic Unit, Plant Sciences Institute, Volcani Center, Agricultural Research Organization, PO Box 6, Bet Dagan 50250, Israel; 2 Department of Vegetable Crops, Newe Ya'ar Research Center, Agricultural Research Organization, PO Box 1021, Ramat Ishai 30095, Israel

## Abstract

**Background:**

Genetic maps constitute the basis of breeding programs for many agricultural organisms. The creation of these maps is dependent on marker discovery. Melon, among other crops, is still lagging in genomic resources, limiting the ability to discover new markers in a high-throughput fashion. One of the methods used to search for molecular markers is DNA hybridization to microarrays. Microarray hybridization of DNA from different accessions can reveal differences between them--single-feature polymorphisms (SFPs). These SFPs can be used as markers for breeding purposes, or they can be converted to conventional markers by sequencing. This method has been utilized in a few different plants to discover genetic variation, using Affymetrix arrays that exist for only a few organisms. We applied this approach with some modifications for marker discovery in melon.

**Results:**

Using a custom-designed oligonucleotide microarray based on a partial EST collection of melon, we discovered 6184 putative SFPs between the parents of our mapping population. Validation by sequencing of 245 SFPs from the two parents showed a sensitivity of around 79%. Most SFPs (81%) contained single-nucleotide polymorphisms. Testing the SFPs on another mapping population of melon confirmed that many of them are conserved.

**Conclusion:**

Thousands of new SFPs that can be used for genetic mapping and molecular-assisted breeding in melon were discovered using a custom-designed oligo microarray. A portion of these SFPs are conserved and can be used in different breeding populations. Although improvement of the discovery rate is still needed, this approach is applicable to many agricultural systems with limited genomic resources.

## Background

Genetic maps based on molecular markers are a key step in marker-assisted selection (MAS) for plant breeding and for gene identification based on positional information [1]. Discovery of novel genetic variation, enhanced selection techniques and the identification of genotypes with new or improved traits can lead to superior strains of interest. The density of the markers on a genetic map defines the map's resolution and the ability to introduce these traits using molecular means. New high-throughput technologies for the discovery of genetic markers and linkage analysis can enhance plant breeding efforts [2].

In the last few years, genomic tools have begun to replace the traditional methods of molecular marker discovery. Use of computational tools to search sequence information for molecular markers [3,4], or genomic technologies such as whole-genome scanning (WGS), have enabled the discovery of thousands of markers in a single experiment [3]. These markers can be mapped to create high-density genetic maps or used directly for MAS [3-5]. However, use of these types of technologies for plant breeding is still restricted, as many agricultural systems have a limited amount of genomic resources. Furthermore, plant species contain wide genetic variation that cannot be explored by sequencing only one or two plants from each species.

One of the technologies that has been used for identification of genetic variation is single-feature polymorphism (SFP) [3-5]. This approach is based on the concept that target DNA that perfectly matches its probes binds with greater affinity than one with a mismatch. Thus, natural imperfections can be detected as a difference in signal intensity in microarray hybridization using labeled genomic DNA (gDNA). SFPs can be used, with no further information, as markers, or they can be sequenced to identify the genetic difference (frequently a single nucleotide polymorphism (SNP) or a small indel). SFP technology draws its strength from the fact that it can be implemented in a variety of genetic applications, such as marker discovery and fine mapping of traits, as well as for genome-wide association studies [3,6,7]. SFP discovery has been relatively successfully implemented in model organisms such as *Arabidobsis *[5] and *Drosophila *[8], and in non-model organisms such as rice and soybean [9,10]. To date, all hybridizations have been performed on high-density short oligonucleotides (Affymetrix arrays). These types of arrays are only available for a few organisms, they are expensive and they are not flexible in their design. Therefore, the ability to implement this technology on any custom array (Agilent, Nimblegen, and others) has the potential to create a very useful tool in many breeding programs for agricultural crops.

Melon is among the most important fleshy fruits for fresh consumption. It belongs to the family Cucurbitaceae which includes other important crops, such as watermelon, cucumber and squash. Melon is a diploid species (2n = 24) of African origin with a high level of phenotypic and molecular variation [11] and a genome size estimated at 450 Mb [12]. Over the last 15 years, several research groups have published genetic maps for melon [13-22]. An integrated map, proposed to serve as a reference map, was constructed, based on two recombinant inbred line (RIL) populations ('Vedrantaise' × PI161375 and 'Vedrantaise' × PI414723). This map contains 668 loci; it spans 1654 cM on 12 linkage groups. It also includes 23 morphological traits [20]. Another map, based on two populations derived from a cross between 'Piel de Sapo' and PI161375, identified several quantitative trait loci (QTLs) controlling fruit-quality traits [18]. The Katzir group has recently established a reference map based on a cross between PI414723 and 'Dulce' [14] and constructed a QTL map using RILs developed from this population [17]. Other resources that were created for melon are a limited EST collection [23] and BAC libraries [24]. Although a substantial amount of effort has been invested in creating genetic maps and molecular tools for melon, only a few genes have been cloned by map-based cloning [25-27]. The genetic maps that exist for melon are not dense enough (containing only a few hundred markers) and are still the limiting factor for MAS, cloning of genes of interest and WGA (genome-wide association studies).

We chose melon as representative of a large group of vegetables and other crops that are agriculturally important but are still lagging behind in their genomic resources. Although sequencing has become extremely affordable using new technologies, we are still not at a stage at which we can sequence a variety of the same plant again and again. In this paper, we present the ability to use a custom-designed microarray with longer oligos (45-55 mer), based on a partial EST database of melon, to discover thousands of new markers for melon. These markers can be used for genetic mapping, breeding and association studies.

## Results

### SFP discovery

We designed a custom oligo microarray (Agilent) based on the melon ESTs presented in the Cucurbit Genomic database (ICuGI) [23]. The microarray contains 186,600 unique probes from 16,114 UniGenes covering approximately 9 Mb of cDNA sequence from different ecotypes and different conditions (for further details, see Methods). For marker discovery, we used DNA from the parents of our mapping population, developed by Katzir's group from a cross between representatives of two subspecies of *Cucumis melo *L.: PI414723 (subspecies *agrestis*) and 'Dulce' (subspecies *melo*). Two biological replicates from PI414723 and two from 'Dulce' were used. Each biological replicate contained gDNAs pooled from 10 different plants. gDNA samples were labeled and hybridized using standard Agilent procedures for comparative genomic hybridization (CGH) (see Methods).

**Figure 1:**
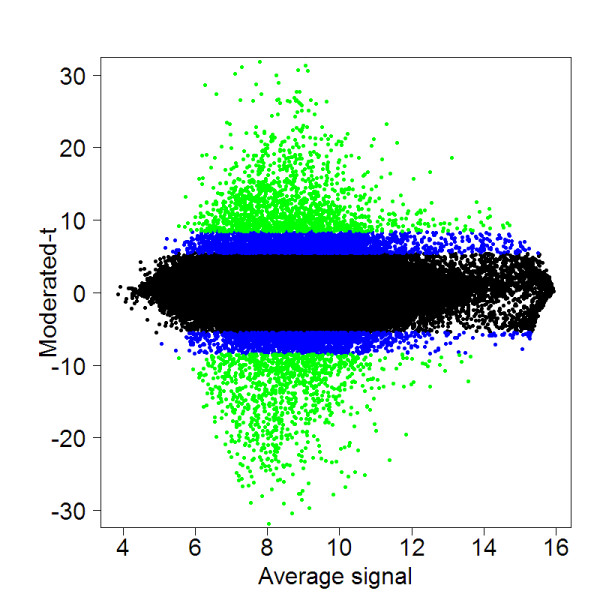
**Genetic variation between two melon accessions**. A scatter plot of moderated-t vs. average signal of four samples (two different plant DNA pools from each accession), where moderated-t is the Bayesian correction of the t-statistic calculated from the comparison between two 'Dulce' biological replicates and two PI414723 biological replicates. Black dots are statistically insignificant differences, i.e., non-SFPs, and blue (adjusted-*p *< 0.05) and green (B ≥ 1.5) dots are statistically significant differences, i.e., putative SFPs.

Predicted SFPs were identified using a linear model for microarray (LIMMA) [28]. The data were analyzed and ranked by two statistics: the adjusted *p*-value (*q*-value) and the B statistic, which calculates the log odds that a given SFP will be different between two populations (Figure 1). The number of SFPs found using an adjusted *p*-value (*q*-value) of 5% was 6184 in 3849 UniGenes. Using the B statistic with B ≥ 1.5, which indicates 82% probability of having differential alleles (SFPs), we found 2503 SFPs in 1771 genes. Due to the fact that the *q*-value and B statistic rank the SFPs in the same order, and since the B statistic requires prior knowledge of the proportion of SFPs while the moderated-t statistic does not, we used the critical value of moderated-t statistic, i.e., a *q*-value < 0.05.

### SFP validation

**Figure 2:**
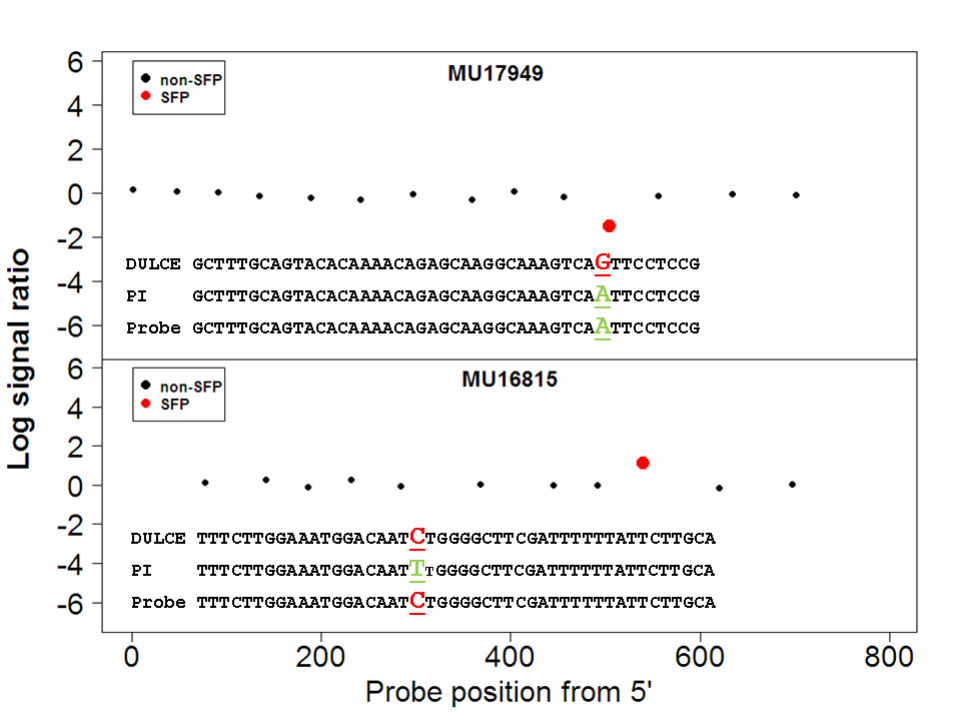
**SFP discovery and validation at the Unigene level**. Examples for genes MU16815 and MU17949 are shown. The graph shows that the signal ratio of the putative SFP is stronger than all other probe sets of the same gene. The signal ratio (y axis) is plotted against the probe position relative to the 5' end of the UniGene contig (x axis). The red dots represent the probes that are predicted to contain SFPs based on statistical analysis. The signal ratio consists of 'Dulce' signal to PI414723 signal, and therefore a positive log signal ratio represents a PI414723 SFP, i.e., 'Dulce' better matches the probe. A negative log signal ratio represents a 'Dulce' SFP. The variation in 'Dulce' and PI414723 SFPs (corresponding to the red dots in the top part of the figure) is revealed by sequencing, illustrated in the bottom part of the graph.

Since the number of available sequences from 'Dulce' and PI414723 in the database is relatively low, we performed validation by direct sequencing of PCR fragments that were amplified from gDNA of the two parents, PI414723 and 'Dulce'. SFPs were chosen randomly for sequencing (with one limitation applied: the flanking regions of the SFPs were set to 50 bp to be able to identify oligos for PCR). Oligos flanking the SFPs were designed and PCR products were amplified from PI414723 and 'Dulce' gDNA pools: 245 putative SFPs were fully sequenced from both plant cultivars. Identification and validation of a SFP is demonstrated in Figure 2. The sequences were compared against the information in the melon database using Vector NTI software (Invitrogen, USA). Most of the SFPs (81%) contained one or two SNPs, and the rest contained either more than two SNPs (13%) or indels (6%) (Additional file 1). Next, we counted the number of true and false positives (TP and FP, respectively) found by sequencing (summarized in Table 1). We found, the most reliable SFPs to be those that appear once per gene. We calculated the positive predictive value (PPV = TP/[TP+FP]) for each number of SFPs per gene category, since the proportion of the genes according to SFP number was biased in our sequencing sample. Our PPV was found to be 79%, similar to that found in rice [29], higher than what has been found in some other SFP-discovery studies, but significantly lower than that which has been found in some studies of *Arabidopsis *and yeast [5,30]. Analysis based on melon EST databases [23] showed that the frequency of variation discovered by our SFP array was 10-fold higher than that found in the partial transcriptome. Another indication that the nucleotide polymorphisms in the detected SFP are biologically true is their polymorphism patterns. Transition nucleotide substitutions are known to be more frequent than transversions [31]. Table 2 summarizes the genotyping as detected by sequencing. The transition-to-transversion ratios in the PI414723 and 'Dulce' populations were 1.6 and 2.5, respectively. Indeed, PI414723, which is expected to be genetically more variable than the domestic cultivar, had a total of 3.23-fold more nucleotide substitutions than 'Dulce' (relative to the database that is composed from different cultivars).

**Table 1:** Sequence validation of putative SFPs

SFP per UniGene	No. of genes	FP	TP	PPV*	Proportion of genes by SFP on array	Proportion of genes by SFP number in sequence validation
1	118	21	97	0.82	0.68	0.55

2	62	17	60	0.78	0.19	0.29

3	21	9	20	0.69	0.07	0.10

>3	12	8	13	0.62	0.06	0.06

Total	213	55	190	*0.79	1	1

Summary of bioinformatics analysis of 245 SFPs sequenced from the two different DNA pools of PI and 'Dulce'. The sequence data were compared to the database data. TP-True positive. FP-False positive. PPV-Positive predictive value.

*Total PPV was recalculated by taking into account the actual representation of the different SFPs per UniGene group in the population. PPV = TP/[TP+FP] for each number of SFPs per gene category. The calculations were performed to adjust the validated SFP results with the proportions discovered by bioinformatics analysis.

**Table 2:** Transition-to-transversion ratios in SFPs

Transversions				Transitions		
**CG<->GC**	**AT<->TA**	**AC<->CA**	**TG<->GT**	**AG<->GA**	**TC<->CT**	

14	21	18	17	53	61	PI414723

2	7	2	5	24	17	'Dulce'

Bioinformatics analysis revealed transitions/transversions by comparing sequence data of SFPs to the melon EST database http://www.icugi.org.

**Figure 3:**
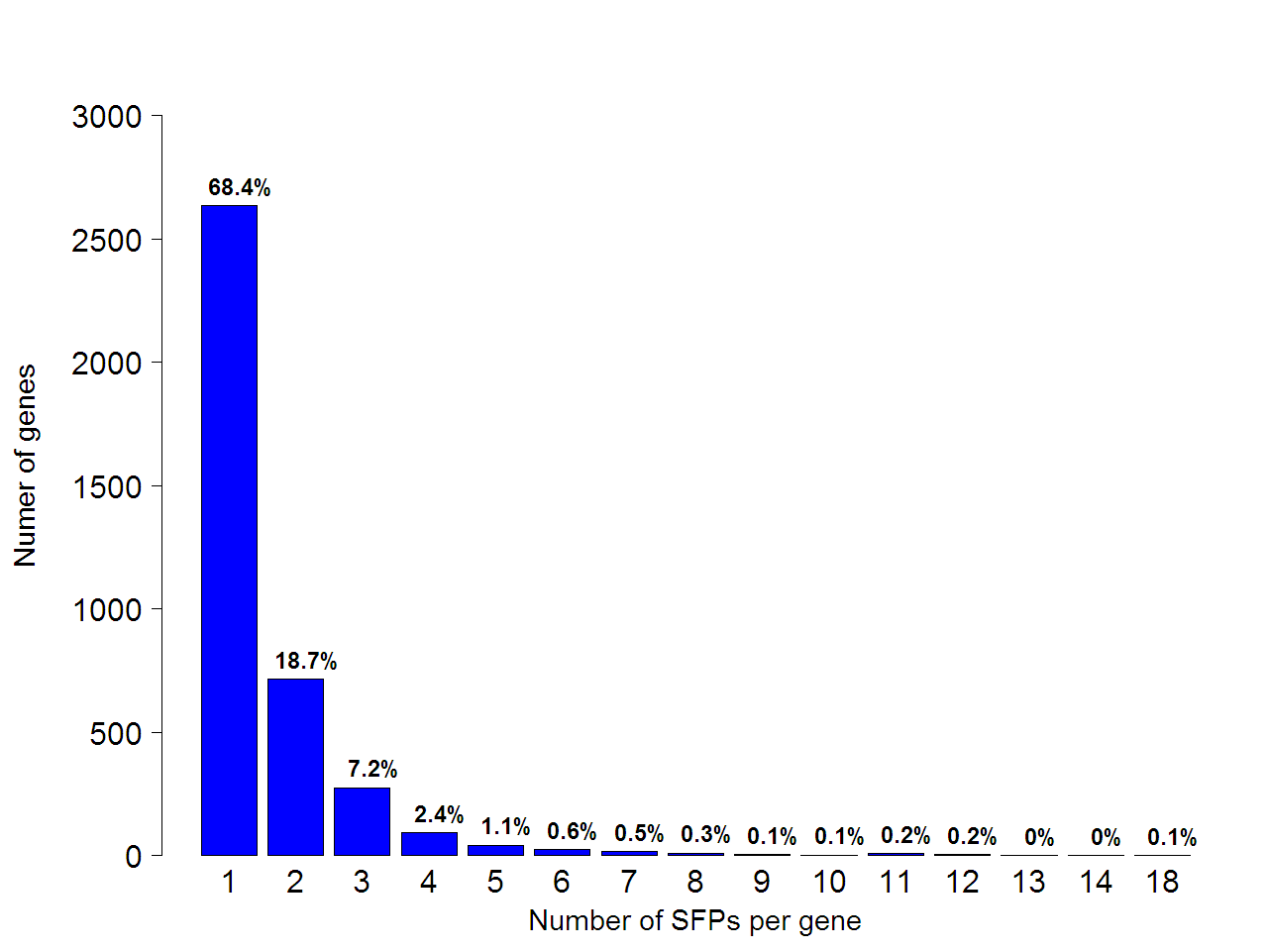
**Distribution of SFPs per gene**. The number of SFPs per UniGene (EST contig) was counted and the frequencies were plotted as a bar plot. Numbers above the columns are the percentage of gene counts in each category out of total counts.

**Figure 4:**
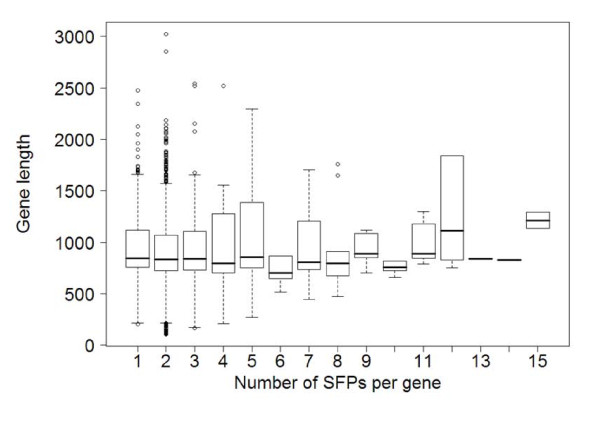
**Number of SFPs per gene is not correlated with UniGene contig length**. Box plots of UniGene contig length for each SFP/gene category. The length of the original contig from which the probes were designed. Descriptive statistics of UniGene length for each SFP/gene category were calculated. Medians of UniGene lengths are represented by horizontal thick lines in the boxes and box borders represent the 25th and 75th percentiles. Medians are approximately the same for all categories.

Based on the distribution of SFPs per gene (Figure 3), most of the genes (~90%) accumulating SFPs possessed one or two SFPs per gene. One might postulate that this is because most of the UniGenes, from which the probes present on the array were designed, are short or partial, and therefore the real number of SFPs per gene cannot be determined. However, the length of the UniGenes is variable and cannot explain the SFP/UniGene variation. For each SFP density category (one SFP per UniGene to 18 SFPs per UniGene), the mean UniGene length was approximately constant at 800 ± 300 bp (Figure 4). In other words, length medians do not increase linearly with SFP per gene category as it would be expected if number of SFPs that were found were due to UniGene length. Therefore, it is reasonable to assume that the SFP/gene distribution was the actual distribution, and it was consistent with the SFP density that has been previously reported for *Arabidopsis *[7].

### Can SFPs be used in different mapping populations?

**Figure 5:**
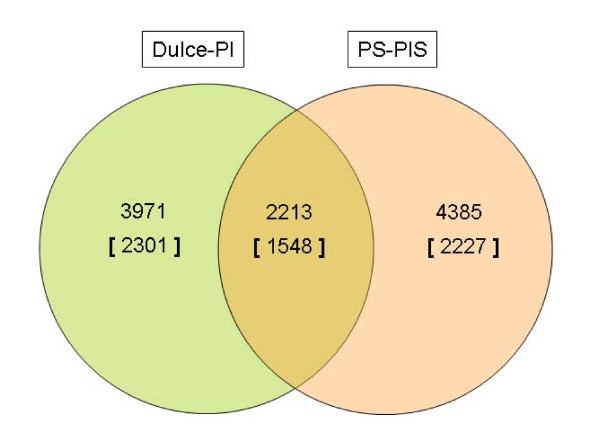
**Conservation of genetic markers between two mapping populations**. Shared SFPs between two mapping populations, PI161375 (PIS)/'Piel de Sapo' (PS) and PI414723 (PI)/'Dulce'. If the same probe was found statistically significant in both comparisons, the SFP was flagged as common. The subset of intersecting SFPs is presented in a Venn diagram. The number of genes including these SFPs is presented in brackets.

**Figure 6:**
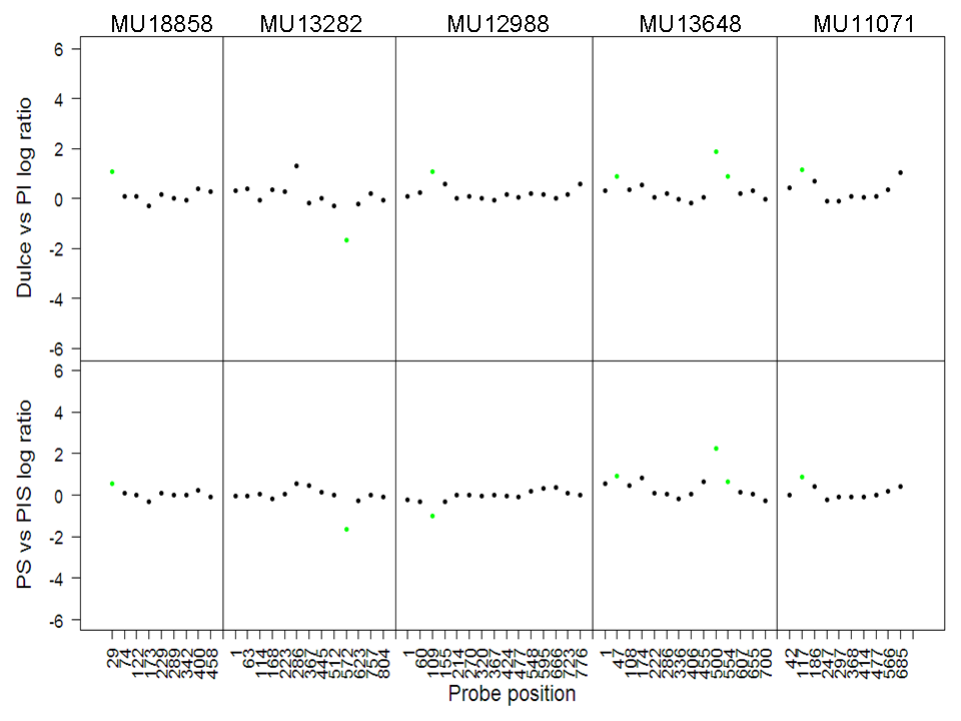
**Conserved sets of SFPs**. An example of five genes with SFPs (green dots) that are conserved between the two mapping populations. Each square is a graph of the log signal ratio between two populations relative to the position of the probe from the 5' end of the UniGene. The five upper squares illustrate the genetic difference between 'Dulce' and PI414723 (PI) and the five lower squares illustrate the genetic difference between PI161375 (PIS) and 'Piel de Sapo' (PS).

The ability of SFPs to function as markers in different genetic backgrounds is crucial for their use in MAS, association studies and genetic mapping. As plants host large genetic diversity, we decided to test the use of a set of SFPs across different melon cultivars. We tested this question using parents of another melon mapping population, 'Piel de Sapo' and PI161375 [18]. We performed hybridizations of two biological replicates (pool of 10 plants) from each genotype on two arrays. The SFPs, i.e., statistically significant signal ratios, that were independently found in both comparisons--'Piel De Sapo' vs. PI161375 and 'Dulce' vs. PI414723 were designated as shared SFPs. In comparisons between the Spanish mapping population's parents, PI161375 and 'Piel de Sapo', and the corresponding genotypes PI414723 and 'Dulce', the number of SFPs was 6598 and 6184 in 3820 and 3849 genes, respectively (Figure 5). Using this set of genetic markers between the two cultivars ('Piel de Sapo' and 'Dulce') and the wild accessions PI414723 and PI161375, we defined the shared SFP set of mapping markers, i.e. the intersection between the 6598 SFPs that differentiate 'Piel de Sapo' from PI161375 and the 6184 SFPs that differentiate 'Dulce' from PI414723. This subset included 2213 SFPs in 1548 UniGenes. An example of five genes from this intersecting subset is illustrated in Figure 6. From the data presented in Figures 5 and 6, we concluded that SFPs can be used across different genetic backgrounds in melon.

## Discussion

In the present study, we tested the feasibility of identifying genetic polymorphism in melon crops by hybridizing gDNA to custom-designed microarrays based on a partial transcriptome. SFP technology has been previously applied for a variety of genetic applications, such as linkage analysis and association studies, mostly in model organisms such as *Arabidopsis *and yeast [5,32], with limited use in some other agricultural organisms [29,33,34]. One of the main reasons for the limited use of this technology is that the technological platform (Affymetrix arrays) is not available for many biological systems. In this study, we used custom-designed arrays with 45-55 mer probes (limitation of the Agilent CGH protocols) covering a substantial area of the partial transcriptome to discover genetic variation in melon. As a result, we increased the number of markers available for melon by an order of magnitude using the cost-effective SFP technology.

The melon cultivars showed substantial within-population genetic variation (Additional file 2), as can be expected in agricultural crops. This needs to be considered when defining the variation between different cultivars. Based on our results, the use of pools of different parents is essential to overcoming this issue.

With some exception [5,32], studies have reported discrepancies between calculated and observed false discovery rates (FDRs) for SFPs. Sequence validation (Table 1) showed that the FDRs in our array (melon, 21%) were similar to those found in rice (19%) [29] and unsequenced *Arabidopsis *strains (15-20%) [33], better than those found in tomato (25-30%) [34], and not as good as those found in some of the *Arabidopsis *studies [5,7]. One reason for this discrepancy is that genetic variation in loci near a target may influence its affinity to probes [35]. In our study, we eliminated all putative SFPs that did not harbor changes inside the SFP, because we could not evaluate the connection between these differences and hybridization to the SFPs. Nevertheless, these differences could still be used as markers. In this work we tried to exclude the possibility of minor frequency alleles (MFAs). For example, focusing on a specific locus, in one parent the allele may be T in its two biological replicates (pools) whereas in the other parent gDNA pools, the T allele might make up only 80% of the pool and G might make up the other 20%. This might affect the hybridization signal, but it would not be observable at the sequence level. By showing that the inter-population variation is greater than the intra-population variation, we exclude this possibility (Additional file 2). Luo et al. [36], using labeled cDNA and Affymetrix array in barley, suggested that many of the SFPs that do not show any differences in direct sequencing are a result of cis-regulatory expression regulators. This explanation is not relevant to our study as we are using genomic DNA for labeling. As only partial transcriptome data exist for melon, the ability to discover SFPs and to eliminate cross-hybridizations and gene duplications is limited. Divergence of exon-intron boundaries and alternative splicing of cDNA that are printed on the array and the labeled genomic DNA could also contribute to the high FDR that is found by sequencing. Another explanation for the discrepancy in FDR can be the power of the statistical test, which is might be too low given the number of replicates in the different experimental systems, We do not believe that this is the reason for the high FDR as we used the empirical Bayesian model that can repair low power in limma [28]). One can improve the discovery rate by ignoring all multiple SFPs (four or more), which can be considered copy number variations (Figure 2). It is also possible that by using this type of long probe array, we lose a portion of the variation; however in doing so, we gain better coverage of the transcriptome, flexibility in format design and lower price. We anticipate that by improving the array design and adding the complementary strand for each probe, the FDR will be reduced as suggested by Smemo et al [37]. As the new Agilent and Nimblgen platforms hold much larger probes sets, one can increase the number of probes for any given sequence without hampering coverage. Another solution is increasing the statistical threshold in order to the decrease the FDR. There no doubt that this approach will reduced the discrepancy in FDR but will also will reduced dramaticly the number of SFPs that are discovered [34].

The question of conservation of the SFP markers across melon cultivars was tested using two different mapping populations (Figures 5 and 6). The data presented here show that a large portion of the SFPs are conserved between the different populations. This experiment demonstrated another advantage of SFP technology, which utilizes large sets of probes with no assumption of the location of the changes in different mapping populations. The large set of probes allows identification of large sets of genetic markers in different mapping populations. Although these sets of markers are not ordered, one can still use them for breeding experiments as a whole -genome screening tool and for creating "classical" genetic maps.

Developing a custom-designed array creates the possibility of using SFP technology in many agricultural systems that do not have an Affymetrix platform and allows much more flexibility in design and use. The low price of the screening and the relatively rapid execution and flexibility make it a useful technology. Although SFP technology is far from perfect, and a reduction in FDRs is needed to make thi approach more efficient, we firmly believe that it can contribute to marker discovery, MAS and WGS approaches in various crop plants. We therefore envision the ability to use WGS (SFP technology and others) as a routine element in the process of MAS in breeding programs as the next challenge in the field of breeding technology.

## Conclusion

SFPs can be discovered using custom-designed microarrays and partial genomic data. This approach can be used with many agricultural organisms for marker discovery and breeding.

## Methods

### Array design

A single-feature array was designed based on 16,637 non-redundant cDNA sequences of the Melon Genomics Database from different genotypes and conditions [23]. This set of sequences was used as input for OligoWIZ 2.0 [38] to design an optimal set of probes along the melon partial transcriptome. We designed probes of 50 base pairs (bp) in length ± 5 bp with T_m _optimization (76.6°C). The T_m_, as well as other features of the probes such as cross-hybridization, folding, and low complexity, were summed to total scores with weightings of 0.245, 0.61, 0.025, and 0.12, respectively. The output of this process was 198,500 probes. Probes with a total score lower than 0.3 that included "N" in their sequence were filtered out, leaving a total of 186,600 probes. This design was loaded on the Agilent eArray site for array production https://earray.chem.agilent.com/earray/.

### Genomic DNA preparation

Genomic DNA was extracted using standard procedures [39] from young leaves pooled from 10 plants of each of the different cultivars. Two separate pools were created for each cultivar. The DNA was treated with RNAseH (Sigma, USA) and re-purified using phenol/chloroform/isoamyl alcohol followed by ethanol precipitation.

### Microarray labeling, hybridization and scanning

gDNA (2.5 μg) was digested with restriction enzymes *Alu*I and *Rsa*I (Promega, USA). The digested DNA was used for labeling and hybridization following Agilent procedures for CGH [40]. Scanning of the array was performed with the Agilent scanner. Labeling, hybridization and scanning were done at the Weizmann Institute's DNA Microarray Facility (Rehovot, Israel).

### Statistical analysis

Probe signal pre-processing and fitting were performed with the R-package for LIMMA [28], following background subtraction; for within-array normalization we applied the "lowess" method [41], and for between-array normalization the "Aquntile" method [42]. To select for statistical significance of the signal ratios, two subsequent steps were applied.

1) A least-square fitting using the following equation:(1)

Where the vector of P-D, D-P, D-D, and P-P is the vector of log signal ratios, the matrix of 1, 0, and -1 is the design matrix, and the vector of D2-D1, P1-D1, and P2-D1 is a vector of the estimated coefficients. Next we used these coefficients to estimate three comparisons (contrasts):

the inter-variation(2)

the intra-PI variation(3)

the intra-'Dulce' variation(4)

2) The ordinary t-statistic of each comparison (contrast) for each SFP probe was moderated by shrinking its standard error towards a common value borrowed from the gene ensemble by Bayesian as described in Smyth 2004 [28]. Therefore, from equations (2), (3), and (4) we used the moderated-t statistics for the statistical significance test. The *p*-value was corrected using Benjamini and Hochberg's method [43] to get an adjusted *p*-value (or *q*-value).

The B statistic is a Bayesian view of the *p*-value giving the log odds that an SFP is true (probe signal ratio is significant). The exact formula of B is described in [44].

### SFP validation

SFPs were selected randomly for validation from the list of putative SFPs. Based on sequence information from the melon database, primers were designed from 50-100 bp of the SFP using Primer3 software [45]. PCRs were performed from DNA pools of PI414723 and 'Dulce' (PCR conditions: 30 cycles of 30 s at 94°C, 30 s at 58°C, 1 min at 72°C). Sequencing was executed by Macrogen Inc., Korea. Sequence analysis was performed using Vector NTI software (Invitrogen). Only samples that showed good-quality sequences from the two parents were analyzed and compared to the database sequence that was used as a reference.

## Abbreviations

SFP: single-feature polymorphism; PPV: positive predictive value; MAS: marker-assisted selection; FDR: false discovery rate; BAC: bacterial artificial chromosome; WGS: whole-genome scanning; gDNA: genomic DNA.

## Authors' contributions

AS and RO conceived the idea of exploring melon variation using custom-designed microarrays. RO designed the array and performed all of the statistical analyses. NK helped in integrating the experimental design for melon biology. VP, RB, YB and GT created the biological resources and did some of the wet work for validation of the results. SU was involved in the bioinformatics analysis of the array data and validation of the results. AS and RE contributed to the experimental design and testing of the microarray output. All authors read and approved the final manuscript.

## Supplementary Material

Additional file 1 **List of 245 positive SFPs and their genotyping**. The table describes the validation by Sanger sequencing and the difference, where there is one, between the probe and targets. Header descriptions. ProbeName - Probe index. Target - Target resource index as in the melon database v2 http://www.icugi.org/. Sequnce - probe sequence. Position - position of genetic variation within probe sequence. SFP - Either 1 for true positive (TP) or 0 for false positive (FP). NoSNP - number of single-nucleotide polymorphisms (SNPs) in the SFP; 999 indicates many. IndelLength - Indel length if one exists. Pi - PI414723 genotype. Dulce - 'Dulce' genotype. Ref - probe genotype. t - the moderated-t statistic. logFC - log2 of PI-to-'Dulce' signal ratio. adj.P.Val - *p*-value after multiple-tests correction by Benjamini and Hochberg method Click here for file

Additional file 2 **Qualitative estimation of the intra-variation within Cucumis *melo *L.: PI414723 and 'Dulce' accessions**. The document includes description of the experimental design and figure of comparison between two melon accessions, PI414723 and 'Dulce', intra- and inter-variation. Click here for file
