# Supplementary material for: High-throughput marker discovery in melon using a self-designed oligo microarray
Source: BMC Genomics. 2010 Apr 28;11:269. doi: 10.1186/1471-2164-11-269 (PMC2874814; doi:10.1186/1471-2164-11-269)
Supplement: Additional file 2 — Qualitative estimation of the intra-variation within Cucumis melo L.: PI414723 and 'Dulce' accessions. The document includes description of the experimental design and figure of comparison between two melon accessions, PI414723 and 'Dulce', intra- and inter-variation. [file 1471-2164-11-269-S2.DOC]

**Qualitative estimation of the intra-variation within *Cucumis melo* L.: PI414723 and ‘Dulce accessions.**

We designed a custom oligo microarray (Agilent) based on the melon ESTs presented in the Cucurbit Genomic database (ICuGI) [25]. The microarray contains 186,600 unique probes from 16,114 UniGenes covering approximately 9 Mb of cDNA sequence from different ecotypes and different conditions (for further details, see Methods).

For marker discovery, we used DNA from the parents of our mapping population, developed by Katzir's group from a cross between representatives of two subspecies of *Cucumis melo* L.: PI414723 (subspecies *agrestis*) and 'Dulce' (subspecies *melo*). Two biological replicates from PI414723 and two from 'Dulce' were used. Each biological replicate contained gDNAs pooled from 10 different plants. gDNA samples were labeled and hybridized using standard Agilent procedures for comparative genomic hybridization (CGH) (see Methods). The inter-population genetic variation was detected by using two arrays, with one of the biological replicates of 'Dulce' against one of the PI414723 replicates on each array. The intra-population variation was detected by hybridizing the PI414723 replicates against each other and the 'Dulce' replicates against each other. Intra-population variation was estimated to ensure that the genetic variation between these populations is based on alleles that are fixed in the population and not due to intra-population variation. In figure 1 we superimposed the differential signal between two pools from the same parental population (intra-variation) over the differential signal that comes from average of pools from one parent against average of pools from the other parent (modified as the moderated t-statistic). Testing the two parents' replicates against themselves showed much greater internal variation in the wild accession PI414723 (Figure 1B) than in the domestic cv. 'Dulce' (Figure 1A), as expected from their genetic backgrounds. Moreover, it was clear that the inter-population variation was greater than the intra-population variation in either PI414723 or 'Dulce'. Thus, we confirmed that the SFPs detected by hybridization between these two populations are a result of fixed alleles in the populations rather than of internal variation.

**Figure 1. Genetic variation between and within mapping populations**. A scatter plot of moderated-t vs. average signal of four samples (two replicates of each population), where moderated-t is the Bayesian correction of the t-statistic calculated from the comparison between two 'Dulce' biological replicates and two PI414723 biological replicates. For both graphs, A and B, black dots are statistically insignificant differences, i.e., non-SFPs, and blue (adjusted-*p* < 0.05) and green (B > 1.5) dots are statistically significant differences, i.e., putative SFPs. The intra-population variation is superimposed as orange dots: (A) internal variation of 'Dulce', and (B) internal variation of PI414723.

The calculation and linear model for this figure is described in the paper material and methods section.
